# Thinner femoral cortical thickness in patients with destructive rheumatoid arthritis of the knee

**DOI:** 10.1186/s13018-023-04340-0

**Published:** 2023-11-09

**Authors:** Rika Kakutani, Naoki Kondo, Go Yamako, Tomoharu Mochizuki, Keiichiro Someya, Hiroyuki Kawashima

**Affiliations:** 1 Division of Orthopedic Surgery, Department of Regenerative and Transplant Medicine, Niigata University Graduate School of Medical and Dental Sciences, 1-757, Asahimachi Dori, Chuo-ku, Niigata, 951-8510 Japan; 2 Department of Mechanical Engineering, Faculty of Engineering, University of Miyazaki, Miyazaki, Japan

**Keywords:** Body mass index, Bone mineral density, Computed tomography, Femoral cortical thickness, Rheumatoid arthritis

## Abstract

**Background:**

The examination of femoral cortical bone thickness in patients with rheumatoid arthritis (RA) has been notably limited in prior research. We aimed to compare femoral cortical thickness in patients with rheumatoid arthritis (RA) and healthy controls and to investigate the association between femoral cortical thickness and clinical parameters within the RA group.

**Methods:**

Forty-four patients (58 limbs) with RA who underwent total knee arthroplasty were enrolled. Preoperative computed tomography images of the lower limbs were analyzed. The femoral cortex was divided into the proximal, central, and distal diaphysis regions and further into the anterior, posterior, medial, and lateral regions. The divisions were measured using Stradwin® software and standardized by femoral length. Femoral cortical thickness was compared between RA and healthy control (*n* = 25) groups. Correlation analyses between standardized cortical thickness and disease parameters were performed in the RA group.

**Results:**

The RA group had significantly lower standardized femoral cortical thickness at the anterior and medial distal diaphysis than healthy controls. Standardized proximal lateral and central lateral in the RA group were significantly larger than those in the healthy control groups. Standardized femoral cortical thickness was significantly correlated with bone mineral density (BMD) in 11 areas, except the posterior central diaphysis, and with body mass index in 8 areas, except the central posterior, distal lateral, distal anterior, and distal medial diaphysis.

**Conclusions:**

Femoral cortical thinning was noted in patients with RA complicated with destructive knee, particularly at the anterior and medial distal diaphysis. Femoral cortical thickness was significantly correlated with BMD and body mass index (BMI); thus, patients with RA and low BMD and BMI should be cared for to prevent fragility fractures.

## Background

Rheumatoid arthritis (RA) is an autoimmune inflammatory disease with synovitis as its predominant pathology [1]. It is characterized by chronic polyarthritis and progressive joint destruction and is most common in women aged 20–50 years [1]. Patients with RA have low bone mineral density (BMD), a frequent cause of fragility fractures [2, 3]. Furthermore, disease duration, cumulative RA disease activity (disease activity score 28 C-reactive protein [CRP]; Disease Activity Score-28 CRP-3 [DAS28-CRP3]), and health assessment questionnaire (HAQ) scores are also associated with fracture risk [4].

Osteoporotic bones have been evaluated using the cortical width on a simple radiograph by measuring the cortical bone of the distal radius [5, 6]. However, bone strength is defined by BMD and bone quality, which includes structural and material characteristics of the trabecular and cortical bone [7, 8]. Furthermore, the quantitative measurement of femoral cortical bone is more accurate using computed tomography (CT) as a base, as established by Treece et al. [9, 10]. Someya et al. also used this method to analyze and report femoral cortical bone width in healthy subjects of different sexes and ages [11]. However, femoral cortical bone thickness in patients with RA has rarely been examined.

The objectives of this study were: 1) to measure the femoral cortical bone thickness of patients with RA quantitatively via CT-based software; 2) to compare the femoral cortical bone thickness of patients with RA with that of healthy patients; and 3) to analyze the correlation between femoral cortical bone thickness in patients with RA and BMD and each disease parameter. We hypothesized that femoral cortical thickness in patients with RA is significantly thinner than that in healthy controls and is significantly correlated with BMD.

## Methods

### Patient registration

Forty-four female patients (58 limbs) who underwent total knee arthroplasty (TKA) in our department between April 2011 and December 2020 for RA destruction of the knee joint were included in the study. Preoperative full-length CT of the lower extremities was performed on the affected and contralateral legs and used for analysis. The healthy group consisted of female volunteers who participated as healthy elderly subjects in the study by Someya et al. (25 limbs in 25 patients) [11]. They were not obese and had no history of trauma or other diseases, such as metabolic bone diseases, except for primary osteoporosis.

The inclusion criteria for the registration were as follows: 1) subjects fulfilled the RA diagnostic criteria, 2) CT examination of the ipsilateral femora is performed for the preoperative planning of TKA. The exclusion criterion was 1) subjects with complications, including ipsilateral femoral fractures and malignancy.

### Measurement methods

**Fig. 1:**
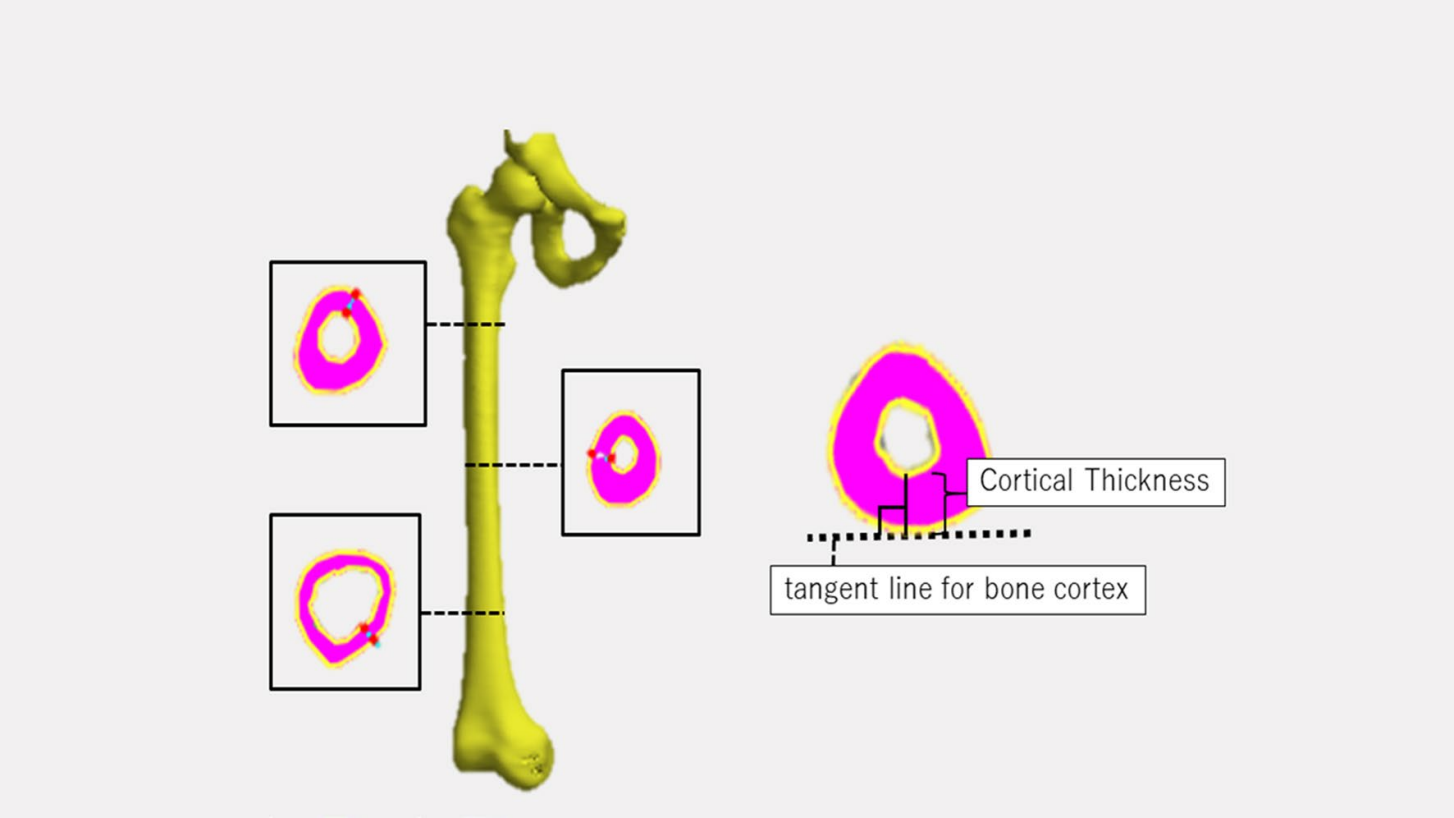
Schematic illustration of the three-dimensional (3D) femoral bone models and femoral cortical thickness mapped back onto the surface as a color. Using the cortical mapping technique, cortical thickness is mapped across the entire surface of the femur. The number of measurement points per femur is 5000–8000 (depending on the length of the femur)

Digital imaging and communications in medicine data from preoperative full-length CT images of the lower extremities were analyzed, and measurement sites and cortical bone thickness were standardized using Someya et al.’s method [11] (Fig. 1); Stradwin® software was used to measure cortical bone thickness. Then, the three-dimensional (3D) femur model was constructed, and the femoral axis was defined as the z-axis. The approximate spheres of the femoral lateral epicondyle and medial epicondyle were determined, and the line connecting the center of each approximate sphere was defined as the x-axis. Finally, the axis perpendicular to the z- and x-axes was defined as the y-axis, and the midpoint of the approximate spheres was the origin of the coordinates.

**Fig. 2:**
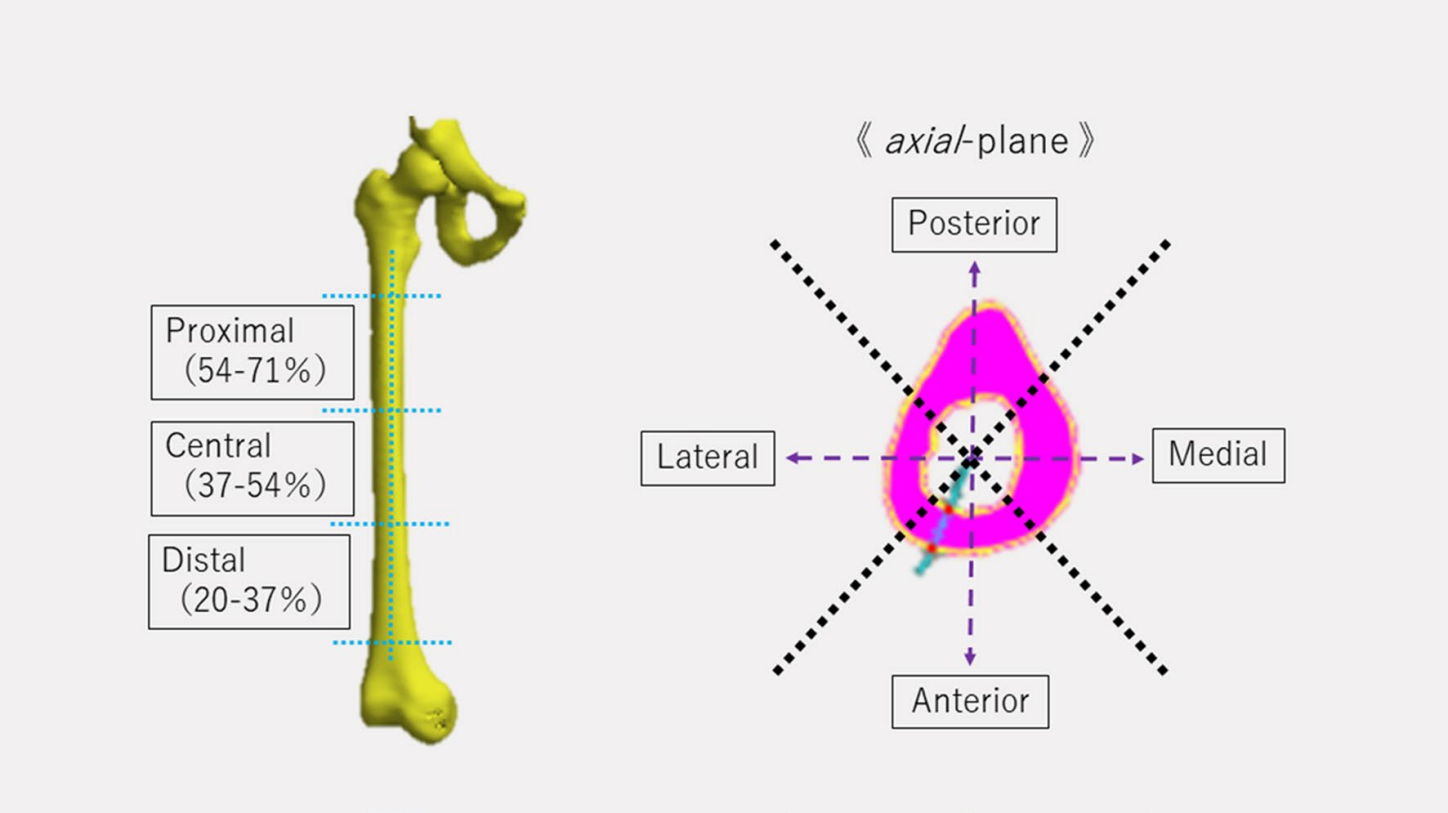
Schematic illustration of the femoral coordinate system and 12 regions divided by each of the three heights (proximal, central, and distal) and 4 areas (medial, anterior, lateral, and posterior). Using principal component analysis, the axis of femur diaphysis was defined as the z-axis. The approximated spheres representing the medial and lateral posterior femoral condyle were determined. The axis connecting the centers of the medial and lateral approximated posterior condylar spheres was defined as the x-axis. The axis perpendicular to the z- and x-axes was defined as the y-axis. The total 12 assessment regions were produced by combining three heights (proximal, 54–71%; central, 37–54%; distal, 20–37%) and four areas of the axial plane (xy-plane) at 90° (medial, anterior, lateral, and posterior). Each region had 200–800 points from the cortical thickness data

The distance from the origin to the femoral head was defined as 100% of the femoral length, and cross-sections parallel to the xy-plane were marked at 20, 3%, 54, and 71% and measured starting from the distal end; the region measured from 20–37% was defined as the distal femur, 37–54% as the mid femur and 54–71% as the proximal femur. Each region was divided into 4 locations, totaling 12: anterior, posterior, medial, and lateral (Fig. 2). Thus, the measurement sites were defined as follows: proximal diaphysis-anterior (PA), proximal diaphysis-posterior (PP), proximal diaphysis-lateral (PL), proximal diaphysis-medial (PM), central diaphysis-anterior (CA), central diaphysis-posterior (CP), central diaphysis-lateral (CL), central diaphysis-medial (CM), distal diaphysis-anterior (DA), distal diaphysis-posterior (DP), distal diaphysis-lateral (DL), and distal diaphysis-medial (DM). Cortical bone thickness was measured exhaustively by averaging 200–800 cross sections in each region (Fig. 2); it was standardized by dividing the measured femoral bone cortical thickness by femoral bone length and evaluated as a standardized cortical bone width value (× 10^−3^).

### Statistical analysis

The Shapiro–Wilk test was used to determine if the femoral cortical bone thickness at each site followed a normal distribution between the RA and healthy control groups. If data were normally distributed, the data were described as mean ± standard deviation and if data were not normally distributed, the data were described as median (first quartile-third quartile).

Then, a t-test was performed for normally distributed data; otherwise, the Mann–Whitney U-test was performed. Pearson's correlation analysis was performed between the 12 standardized cortical bone thickness values and the BMD (T-score; BMD was measured at either the lumbar spine or femoral neck; if more than one site was measured, the lower value was selected), BMI, RA disease duration, serum matrix metalloproteinase-3 (MMP-3) level, rheumatoid factor (RF) value, DAS28-CRP3 (RA disease activity), daily prednisolone (PSL) dose, and weekly methotrexate (MTX) dose in the RA group. Thesoftware SPSS version 25.0 (IBM Corp.: Chicago, IL, USA) was used for statistical analyses, and *p* < 0.05 was defined as a statistically significant difference.

### Institutional review board

The study was approved by the Ethics Committee of Niigata University School of Medicine (IRB#2018–0377), and written informed consent was obtained from enrolled patients.

## Results

### Demographic data (Table 1)

**Table 1:** Comparison of subjects in RA and healthy control groups

	Unit	RA group	Healthy control group		*p* value
Number of subjects	cases (limbs)	44(58)	25(25)		
Age	years old	67 ± 7.2	68 ± 5.0		N.S
Body mass index	kg/m^2^	24.0 ± 4.7	23.7 ± 1.2		N.S
RA disease durations	Years	14.0(6.37–24)			
Rheumatoid factor	IU/ml	54.8(15.7–147)			
DAS28-CRP3		2.07(1.76–2.45)			
Bone mineral density (T-score)		− 2.4 (− 3.1– − 1.4)			
MMP-3	ng/ml	147.4 (69.4–362)			
Dose of methotrexate	mg/week	4.0(0–6.0)			
Dose of prednisolone	mg/day	1.0 (0–4.5)			
The treated rate of bDMARDs	cases, %	15, 34.1			
The treated rate of bisphosphonates	cases, %	20, 45.5			

*DAS28-CRP3* Disease Activity Score-28 CRP-3; *MMP-3* matrix metalloproteinase-3; *bDMARDs* biological DMARDs, *n.s.* not significant

Data show mean ± standard deviation if followed the normal distribution. Otherwise, data show median (the 1st quartile- the 3rd quartile), respectively

The mean age was 67 ± 7.2 years old and 68 ± 5.0 years old, and the mean BMI was 24.0 ± 4.7 and 23.7 ± 1.2 in the RA and healthy control groups, respectively, showing no difference between the two groups.

In the RA group, the average duration of RA was 14.0 years (the 1st quartile: 6.37- the 3rd quartile: 24); mean serum rheumatoid factor (RF) level and DAS28-CRP3 were 54.8 (15.7-147.2) IU/ml and 2.07 (1.76-2.45), respectively. The average T-score (lumbar spine or femoral neck) was − 2.4 (-3.1–− 1.4), and serum MMP-3 level was 147.4 (69.4-362) ng/ml. The mean MTX and PSL dosages were 4.0 (0-6.0) mg/week and 1.0 (0-4.5) mg/day, respectively. bDMARDs were used in 15 patients (34.1%), and bisphosphonates were used in 20 patients (45.5%; Table 1). Forty-three and 15 knees were Larsen grades III and IV, respectively.

### The raw values for cortical thickness in the RA and healthy control groups (Table 2)

**Table 2:** Comparison of femoral cortical bone thickness raw values between the RA and healthy groups

	RA group		Healthy control group		
Site of the measurements	Average(minimum–maximum)	Standard deviation	Average(minimum–maximum)	Standard deviation	*p* value
PP	5.24 (2.83–7.14)	1.14	5.69 (4.58–7.15)	0.68	0.069♭
PA	4.91 (3.45–6.98)	0.72	5.13 (4.58–7.15)	0.39	0.03♭
PL	5.69 (4.01–7.98)	0.97	5.43 (4.43–6.94)	0.63	0.216
PM	5.91 (3.51–8.63)	1.06	6.18 (4.86–8.22)	0.89	0.253
CP	5.59 (2.50–7.00)	0.95	6.18 (4.64–7.86)	0.82	0.004 ♭
CA	4.32 (3.05–6.42)	0.7	4.94 (4.35–5.65)	0.35	0.001♭
CL	5.43 (3.87–8.20)	0.94	5.49 (4.47–6.70)	0.49	0.514♭
CM	5.22 (3.16–8.18)	1.03	5.97 (4.89–8.74)	0.84	0.001♭
DP	4.39 (2.58–6.20)	0.68	4.93 (4.23–5.57)	0.36	0.001
DA	3.14 (2.05–6.20)	0.5	4.42 (3.98–4.82)	0.24	0.001
DL	3.95 (2.83–5.79)	0.59	4.50 (4.10–5.01)	0.22	0.001
DM	3.55 (2.49–5.06)	0.6	4.60 (4.20–5.39)	0.3	0.001

♭The Mann–Whitney test was performed. Otherwise, *t* tests were performed

*SD* standard deviation, *PP* proximal posterior, *PA* proximal anterior, *PL* proximal lateral, *PM* proximal medial, *CP* central posterior, *CA* central anterior, *CL* central lateral, *CM* central medial, *DP* distal posterior, *DA* distal anterior, *DL* distal lateral, *DM* distal medial

The mean cortical bone thickness at the proximal femur was as follows (RA vs. healthy controls): 5.24 ± 1.14 (2.83–7.14) mm vs. 5.69 ± 0.68 (4.58–7.15) mm at PP (*p* = 0.069), 4.91 ± 0.72 (3.45–6.98) mm vs. 5.13 ± 0.39 (4.58–7.15) mm at PA (*p* = 0.03), 5.69 ± 0.97 (4.01–7.98) mm vs. 5.43 ± 0.63 (4.43–6.94) mm at PL 8p = 0.216), and 5.91 ± 1.06 (3.51–8.63) mm vs. 6.18 ± 0.89 (4.86–8.22) mm at PM (*p* = 0.253). Cortical bone thickness was significantly thinner in the RA group than in healthy controls only at PA (*p* < 0.05).

The mean cortical bone thickness at the mid femur was as follows (RA vs. healthy controls): 5.59 ± 0.95 (2.50–7.00) mm vs. 6.18 ± 0.82 (4.64–7.86) mm at CP (*p* = 0.004), 4.32 ± 0.70 (3.05–6.42) mm vs. 4.94 ± 0.35 (4.35–5.65) mm at CA (*p* = 0.001), 5.43 ± 0.94 (3.87–8.20) mm vs. 5.49 ± 0.49 (4.47–6.70) mm at CL (*p* = 0.514), and 5.22 ± 1.03 (3.16–8.18) mm vs. 5.97 ± 0.84 (4.89–8.74) mm at CM (*p* = 0.001). Cortical bone thickness was significantly thinner in the RA group than in healthy controls at CP (*p* < 0.01), CA (*p* < 0.001), and CM (*p* < 0.001).

The mean cortical bone thickness at distal femur was as follows (RA vs. healthy controls): 4.39 ± 0.68 (2.58–6.20) mm vs. 4.93 ± 0.36 (4.23–5.57) mm at DP, 3.14 ± 0.50 (2.05–4.68) mm vs. 4.42 ± 0.24 (3.98–4.82) mm at DA, 3.95 ± 0.59 (2.83–5.79) mm vs. 4.50 ± 0.22 (4.10–5.01) mm at DL, and 3.55 ± 0.60 (2.49–5.06) mm vs 4.60 ± 0.30 (4.20–5.39) mm at DM. Cortical bone thickness was significantly thinner in the RA group than in healthy controls at DP, DA, DL, and DM (*p* = 0.001).

The mean standardized values for cortical bone thickness at the proximal femur were as follows (RA vs. healthy controls): 14.56 ± 3.15 (7.46–20.66) mm and 14.25 ± 1.53 (11.20–16.99) mm at PP (*p* = 0.636), 13.64 ± 2.03 (9.01–18.31) mm and 12.86 ± 0.86 (11.52–15.00) mm at PA (*p* = 0.063), 15.81 ± 2.79 (10.50–21.79) mm and 13.64 ± 1.68 (11.46–18.46) mm at PL (*p* = 0.001), and 16.43 ± 2.90 (9.17–22.38) mm and 15.48 ± 2.03 (12.17–20.29) mm at PM (*p* = 0.126). Cortical bone thickness was significantly thicker in the RA group than in healthy controls at PL (*p* = 0.001; Table 3).

**Table 3:** Comparison of the standardized values of femoral cortical bone thickness between the RA and healthy groups

	RA group		Healthy control group		
Site of the measurements	Average(minimum–maximum)	Standard deviation	Average(minimum–maximum)	Standard deviation	*p* value
PP	14.56 (7.46–20.66)	3.15	14.25 (11.20–16.99)	1.53	0.636
PA	13.64(9.01–18.31)	2.03	12.86 (11.52–15.00)	0.86	0.063
PL	15.81(10.50–21.79)	2.79	13.64 (11.46–18.46)	1.68	0.001♭
PM	16.43 (9.17–22.38)	2.9	15.48 (12.17–20.29)	2.03	0.126
CP	15.25 (7.16–21.18)	2.66	15.49 (12.15–19.35)	1.89	0.983♭
CA	11.97 (7.96–16.61)	1.81	12.40(11.36–14.21)	0.77	0.25
CL	14.91(1.75–20.32)	3.01	13.78(11.67–18.71)	1.43	0.037♭
CM	14.49 (8.24–20.02)	2.73	14.97 (12.76–21.58)	1.91	0.415
DP	12.20 (7.39–16.79)	1.95	12.37 (11.05–13.70)	0.78	0.667
DA	8.71 (5.93–13.42)	1.34	11.11 (9.50–12.61)	0.71	0.001
DL	10.99(7.90–15.88)	1.75	11.31(10.09–13.80)	0.83	0.380 ♭
DM	9.86 (6.88–14.31	1.67	11.55(10.52–13.68)	0.79	0.001♭

The measured femoral cortical bone thickness (mm) divided by the femoral length (mm) was multiplied by 1000, and the value was defined as the standardized value

♭: the Mann–Whitney test was performed. Otherwise, *t* tests were performed

*SD* standard deviation, *PP* proximal posterior, *PA* proximal anterior, *PL* proximal lateral, *PM* proximal medial, *CP* central posterior, *CA* central anterior, *CL* central lateral, *CM* central medial, *DP* distal posterior, *DA* distal anterior, *DL* distal lateral, *DM* distal medial

The mean standardized values for cortical bone thickness at the mid femur were as follows (RA vs. healthy controls): 15.25 ± 2.66 (7.16–21.18) mm and 15.49 ± 1.89 (12.15–19.35) mm at CP (*p* = 0.983), 11.97 ± 1.81 (7.96–16.61) mm and 12.40 ± 0.77 (11.36–14.21) mm at CA (*p* = 0.25), 14.91 ± 3.01 (1.75–20.32) mm and 13.78 ± 1.43 (11.67–18.71) mm at CL(*p* = 0.037), and 14.49 ± 2.73 (8.24–20.02) mm and 14.97 ± 1.91 (12.76–21.58) mm at CM (*p* = 0.415). Cortical bone thickness was significantly thicker in the RA group than in healthy controls at CL (*p* < 0.05; Table 3).

The mean standardized values for cortical bone thickness at the distal femur were as follows (RA vs. healthy controls): 12.20 ± 1.95 (7.39–16.79) mm and 12.37 ± 0.78 (11.05–13.7) mm at DP (*p* = 0.667), 8.71 ± 1.34 (5.93–13.42) mm and 11.11 ± 0.71 (9.50–12.61) mm at DA (*p* = 0.001), 10.99 ± 1.75 (7.90–15.88) mm and 11.31 ± 0.83 (10.09–13.8) mm at DL (0.380), and 9.86 ± 1.86 (6.88–14.31) mm and 11.55 ± 0.79 (10.52–13.68) mm at DM (*p* = 0.001). Cortical bone thickness was significantly thinner in the RA group than in healthy controls at DA and DM (*p* = 0.001; Table 3).

In summary, compared with the healthy control group, standardized PP and CP were significantly thicker, and standardized DA and DM were significantly thinner in the RA group.

The correlation analysis between the standardized femoral cortical thickness and each clinical parameter in patients with RA is shown in Table 4.

**Table 4:** Correlation between femoral cortical bone thickness and each parameter in the RA group

Site of the measurements	T-score	BMI	RA disease duration	MMP-3	RF	DAS28-CRP3	PSL dose	MTX dose
PP	0.296**	0.294**	− 0.164	− 0.072	− 0.141	− 0.213*	0.011	0.314**
	(*p* = 0.008)	(*p* = 0.006)	(*p* = 0.116)	(*p* = 0.533)	(*p* = 0.194)	(*p* = 0.049)	(*p* = 0.921)	(*p* = 0.003)
PA	0.554**	0.281**	− 0.033	0.208	− 0.127	− 0.192	0.106	0.186
	(*p* = 0.001)	(*p* = 0.009)	(*p* = 0.716)	(*p* = 0.068)	(*p* = 0.245)	(*p* = 0.076)	(*p* = 0.330)	(*p* = 0.086)
PL	0.460*	0.214*	− 0.086	0.156	− 0.129	− 0.261*	0.129	0.185
	(*p* = 0.001)	(*p* = 0.048)	(*p* = 0.394)	(*p* = 0.173)	(*p* = 0.238)	(*p* = 0.015)	(*p* = 0.236)	(*p* = 0.088)
PM	0.556*	0.271*	− 0.051	0.067	− 0.081	− 0.265*	0.114	0.14
	(*p* = 0.001)	(*p* = 0.012)	(*p* = 0.578)	(*p* = 0.561)	(*p* = 0.460)	(*p* = 0.014)	(*p* = 0.297)	(*p* = 0.198)
CP	− 0.130	0.161	− 0.131	0.01	− 0.008	− 0.032	− 0.048	0.279**
	(*p* = 0.251)	(*p* = 0.139)	(*p* = 0.204)	(*p* = 0.931)	(*p* = 0.944)	(*p* = 0.772)	(*p* = 0.662)	(*p* = 0.009)
CA	0.484**	0.304**	− 0.143	0.001	− 0.103	− 0.261*	0.187	0.213*
	(*p* = 0.001)	(*p* = 0.004)	(*p* = 0.180)	(*p* = 0.995)	(*p* = 0.345)	(*p* = 0.015)	(*p* = 0.085)	(*p* = 0.049)
CL	0.593**	0.336**	− 0.255	0.145	− 0.115	− 0.169	0.082	0.158
	(*p* = 0.001)	(*p* = 0.002)	(*p* = 0.104)	(*p* = 0.206)	(*p* = 0.292)	(*p* = 0.120)	(*p* = 0.455)	(*p* = 0.147)
CM	0.595**	0.339**	− 0.213*	− 0.059	− 0.254*	− 0.304**	0.189	0.183
	(*p* = 0.001)	(*p* = 0.001)	(*p* = 0.049)	(*p* = 0.606)	(*p* = 0.019)	(*p* = 0.004)	(*p* = 0.081)	(*p* = 0.092)
DP	0.416**	0.250*	− 0.071	0.280*	0.013	0.063	0.015	0.096
	(*p* = 0.001)	(*p* = 0.021)	(*p* = 0.471)	(*p* = 0.013)	(*p* = 0.906)	(*p* = 0.566)	(*p* = 0.890)	(*p* = 0.379)
DA	0.313**	0.086	− 0.157	− 0.153	− 0.13	− 0.015	0.113	0.332**
	(*p* = 0.005)	(*p* = 0.431)	(*p* = 0.130)	(*p* = 0.181)	(*p* = 0.234)	(*p* = 0.891)	(*p* = 0.299)	(*p* = 0.002)
DL	0.463**	0.193	− 0.162	0.171	− 0.159	− 0.099	− 0.026	0.12
	(*p* = 0.001)	(*p* = 0.074)	(*p* = 0.092)	(*p* = 0.134)	(*p* = 0.144)	(*p* = 0.363)	(*p* = 0.812)	(*p* = 0.271)
DM	0.462**	0.198	0.114	0.143	− 0.229*	− 0.134	0.127	0.112
	(*p* = 0.001)	(*p* = 0.067)	(*p* = 0.254)	(*p* = 0.213)	(*p* = 0.034)	(*p* = 0.219)	(*p* = 0.244)	(*p* = 0.303)

*PP* proximal posterior, *PA* proximal anterior, *PL* proximal lateral, *PM* proximal medial, *CP* central posterior, *CA* central anterior, *CL* central lateral, *CM* central medial, *DP* distal posterior, *DA* distal anterior, *DL* distal lateral, *DM* distal medial

There was a positive correlation between the T-score and the standardized femoral cortical thickness in all the measurement sites except for CP; stronger positive correlations were noted at PA (*r* = 0.554, *p* < 0.001), PM (*r* = 0.556, *p* < 0.001), CL (*r* = 0.593, *p* < 0.001), and CM (*r* = 0.595, *p* < 0.001). There were also positive correlations between BMI and the standardized femoral cortical thickness in eight sites (PP, PA, PL, PM, CA, CL, CM, and DP), especially in proximal (PP and PA) and in central (CA, CL, CM; *p* < 0.01).

In addition, positive significant correlations were detected in MTX dose (mg/week) in four sites (PP, CP, CA, and DA; *p* < 0.05). Conversely, DAS28-CRP3 showed significantly negative correlations at PP (*r* = -0.213, *p* = 0.049), PL (*r* = -0.261, *p* = 0.015), PM (*r* = -0.265, *p* = 0.014), CA (*r* = -0.261, *p* = 0.015), and CM (*r* = -0.304, *p* = 0.004). Analysis was also performed for RA disease duration, MMP-3, RF, and PSL daily dose (mg/day), but no significant correlations were found except one site (Table 4).

## Discussion

Studies on cortical bone thickness in patients with RA have been previously published; however, these only focused on the phalanges [5], distal radius [6, 12], and tibia [12]. This study focused on the femur using a 3D femoral diaphysis cortical bone width measurement system (Stradwin® software) [9–11]. We believe our results are accurate and reproducible owing to our use of a 3D model of the femur constructed through CT imaging and the application of a universal coordinate system and consistent measurement method (Figs. 1, 2).

Femoral cortical bone thickness in women with postmenopausal osteoporosis and their finite element analysis showed that a thinner femoral cortical bone increased the risk of diaphyseal fracture [13]. On the other hand, cortical bone thickness at the distal radius was compared between healthy subjects and patients with RA using high-resolution peripheral quantitative CT (HRpQCT), and the RA group had significantly thinner femoral cortices in both men and women than those in healthy subjects [12].

The present study showed that standardized femoral cortical thickness in the RA group had significantly thicker femoral cortical bone at PL than the healthy control group but significantly thinner femoral cortical bone at DA and DM (Table 3). The mechanism that leads to cortical thickening at PL in the RA group is unclear. However, there was no difference at PL between BP-treated and non-BP-treated patients, within the RA group and no atypical femur fractures occurred. Thus, PL is unlikely to be involved in abnormal bone metabolism or atypical femur fractures. Notably, we compared femoral cortical bone thickness between the RA and healthy groups, which were matched for sex, age, and BMI.

Significant thinned femoral cortical thickness in DA and DM could increase the risk of supracondylar femoral fracture after TKA. In a study of 32 patients, supracondylar femoral fractures after TKA were common in patients with osteoporotic bones [14]; these findings are supported by the results of the present study. Furthermore, the third metacarpal cortical thickness ratio, measured manually on a radiograph, was positively correlated with the BMD of the lumbar spine and femoral neck [5]. Additionally, the radial diaphysis cortical thickness in patients with distal radius fractures, measured using dual-energy X-ray absorptiometry, was positively correlated with the femoral neck bone density [15].

In this study, femoral cortical bone thickness had a significant positive correlation for all measurement sites except BMD and CP, similar to these studies [5, 15]. The radial cortex of patients with RA was analyzed using HRpQCT, showing that it was significantly thinner than in the healthy group and inversely correlated with disease activity [16]. Conversely, there was no association between disease activity or HAQ scores and cortical bone thickness of the distal radius in patients with RA [13]. Furthermore, RA does not contribute to reduced BMD if disease activity is controlled in clinical remission [5]. The disease activity of patients with RA (DAS28) was also well-controlled in this study, becases its median value was 2.07 (Table 1). Furthermore, femoral cortical bone thickness was negatively correlated with DAS28-CRP3 in some areas but not DA or DM (Table 4).

To verify whether lower extremity alignment is related to femoral cortical thickness in patients with RA, we further investigated the association between femorotibial angle (FTA) and PP and CP in patients with RA. The average FTA was 176 ± 11.9° (range; 143–200°). No significant association between FTA and both PP and CP was detected. In addition, we could not get FTA data in the healthy control group, so we could not compare these two groups in terms of FTA.

A limitation of this study is that this group of patients presented with severe deformity of the knee joint, which was an indication for prosthesis. There were no comparisons with patients with RA having mild or moderate disease. Additionally, bisphosphonates and other medication use varied for each patient, and the BMD test results were unavailable for the healthy group. Thus, whether these predisposing factors affect femoral cortical bone thickness is unclear. Furthermore, joint pain during weight-bearing may also lead to limited weight-bearing on the affected side, resulting in reduced stress from loading, which can affect cortical bone thickness. Lastly, the mechanisms by which femoral cortical bone thickness is related to disease activity in RA have not been elucidated, suggesting another mechanism for detecting femoral cortical bone thickness that differs from RA disease activity control. Further research is required on the detection of muscle volume, degree of activities of daily living or quality of life, and other factors.

## Conclusions

This study demonstrated the applicability of CT in the quantitative measurement of femoral cortical bone thickness in patients with RA. Femoral cortical bone thickness was significantly lower at DA and DM in the RA group than in the healthy group, showing a significant positive correlation with BMD and BMI. These findings suggest that patients with RA with low BMD and BMI values also have thinned femoral cortical bones and low bone quality. Thus, the prevention of fragility fractures in these patients is important.

## Data Availability

The data that support the findings of this study are available from the corresponding author, NK, upon reasonable request.

## Abbreviations

RA: Rheumatoid arthritis
BMD: Bone mineral density
BMI: Body mass index
CRP: C-reactive protein
DAS28-CRP3: Disease activity score-28 CRP-3
HAQ: Health assessment questionnaire
CT: Computed tomography
TKA: Total knee arthroplasty
PA: Proximal diaphysis-anterior
PP: Proximal diaphysis-posterior
PL: Proximal diaphysis-lateral
PM: Proximal diaphysis-medial
CA: Central diaphysis-anterior
CP: Central diaphysis-posterior
CL: Central diaphysis-lateral
CM: Central diaphysis-medial
DA: Distal diaphysis-anterior
DP: Distal diaphysis-posterior
DL: Distal diaphysis-lateral
DM: Distal diaphysis-medial
MMP-3: Matrix metalloproteinase-3
RF: Rheumatoid factor
PSL: Prednisolone
MTX: Methotrexate
bDMARDs: Biological disease modifying anti-rheumatic drugs
FTA: Femorotibial angle
